# Comprehensive comparative analysis of the prognostic impact of systemic inflammation biomarkers for patients underwent cardiac surgery

**DOI:** 10.3389/fimmu.2023.1190380

**Published:** 2023-08-14

**Authors:** Zhang Liu, Ge Zhu, Yonggui Zhang, Peng Zhang, Wangfu Zang, Zile Shen

**Affiliations:** ^1^ Department of Cardio-Thoracic Surgery, Shanghai Tenth People’s Hospital, School of Medicine, Tongji University, Shanghai, China; ^2^ Department of Gastrointestinal Surgery, Shanghai Tenth People’s Hospital, School of Medicine, Tongji University, Shanghai, China

**Keywords:** systemic inflammation, biomarkers, cardiac surgery, prognosis, lymphocyte-to-C-reactive protein ratio

## Abstract

**Background:**

Inflammation plays an integral role in the development of cardiovascular disease, and few studies have identified different biomarkers to predict the prognosis of cardiac surgery. But there is a lack of reliable and valid evidence to determine the optimal systemic inflammatory biomarkers to predict prognosis.

**Methods:**

From December 2015 and March 2021, we collected 10 systemic inflammation biomarkers among 820 patients who underwent cardiac surgery. Time-dependent receiver operating characteristic curves (ROC) curve at different time points and C-index was compared at different time points. Kaplan–Meier method was performed to analyze overall survival (OS). Cox proportional hazard regression analyses were used to assess independent risk factors for OS. A random internal validation was conducted to confirm the effectiveness of the biomarkers.

**Results:**

The area under the ROC of lymphocyte-to-C-reactive protein ratio (LCR) was 0.655, 0.620 and 0.613 at 1-, 2- and 3-year respectively, and C-index of LCR for OS after cardiac surgery was 0.611, suggesting that LCR may serve as a favorable indicator for predicting the prognosis of cardiac surgery. Patients with low LCR had a higher risk of postoperative complications. Besides, Cox proportional hazard regression analyses indicated that LCR was considered as an independent risk factor of OS after cardiac surgery.

**Conclusion:**

LCR shows promise as a noteworthy representative among the systemic inflammation biomarkers in predicting the prognosis of cardiac surgery. Screening for low LCR levels may help surgeons identify high-risk patients and guide perioperative management strategies.

## 1 Introduction

Cardiac surgery is the therapy of choice to treat complex or three-vessel coronary artery disease, congenital heart defects and valvular heart disease (1). Despite the decade-long effort of surgeons to improve cardiac surgery safety, it remains a high-risk procedure (2). Existing literature offers little guidance on best practices for identifying errors and improving procedure safety. Currently available risk models, such as EuroSCORE II and STS Risk Score, might not account for all prognostically significant risk factors (3). Therefore, it is essential to make every effort to identify additional risk factors and perform a comprehensive preoperative assessment to guide perioperative management strategies.

Systemic inflammation biomarkers are more commonly used in cancer patients, and they have been demonstrated to be predictive of tumor progression and overall survival (OS) (4–6). Systemic inflammation biomarkers take advantage of being non-invasive, inexpensive and easily accessible, and they have the potential to be cost-effective prognostic markers. However, there are few studies on inflammation biomarkers and the prognosis of cardiac surgery. Considering that inflammation plays an integral role in the pathogenesis and the development of cardiovascular disease (CVD) (7–9), it is necessary to assess the inflammatory status of patients underwent cardiac surgery. Moreover, inflammation is not only a causal agent of CVD but also a surrogate prognostic biomarker, implying the feasibility of anti-inflammatory therapy to improve CVD outcomes (10). Therefore, early detection and assessment of patients’ inflammatory status is of great significance in identifying patients with high inflammatory status, thus improving clinical outcomes and guiding anti-inflammatory intervention. Given the lack of reliable and valid evidence to determine the best combination of systemic inflammation biomarkers to predict prognosis, this study aims to compare various combination of systemic inflammation biomarkers systematically and comprehensively to identify the optimal systemic inflammation markers to assess prognosis in patients underwent cardiac surgery. To our knowledge, this is the first study to report the prognostic value of lymphocyte-to-C-reactive protein ratio (LCR) in patients underwent cardiac surgery. We also conducted a randomized internal validation to confirm the reliability of the conclusions.

## 2 Methods

### 2.1 Participants

This study encompassed patients who underwent coronary artery bypass grafting (CABG) and/or valve surgery at Shanghai Tenth People’s Hospital between December 2015 and March 2021. Adult patients (≥18 years) who possessed complete measurements of serum systemic inflammatory markers and received first major cardiac surgery through midline sternotomy met the inclusion criteria of the study. Aspirin antiplatelet therapy was routinely discontinued 5 days prior to surgery. Antibiotics were also not administered preoperatively. The exclusion criteria were as follows: (1) patients in emergency surgery or admitted to intensive care unit at the start of recruitment; (2) patients with significant clinical evidence of infection or inflammation; (3) cancer patients; (4) patients with incomplete data on peripheral systemic inflammatory features; (5) patients who are unable to communicate or provide informed consent. This project was registered at http://www.chictr.org.cn (registration number: ChiCTR2200056468) and ethical approval was obtained from the Ethics Review Committee of Shanghai Tenth People’s Hospital. All participants signed a written informed consent to participate in this study.

### 2.2 Baseline data collection

For each patient, demographic information was acquired by researchers trained in the project on admission, including age, sex, height, weight, comorbidities and lifestyle (smoking and drinking). During hospitalization, disease- and treatment-related information was recorded for all patients, including surgical type, operative time, postoperative complications (classified on the basis of the Clavien-Dindo classification), length of hospitalization, hospitalization expenses, and readmissions. Only complications categorized as grade II or higher were analyzed.

### 2.3 Measurements of serum systemic inflammation biomarkers

Blood samples were collected from each patient within 48 hours prior to receiving surgery and serological information was tested, including white blood cells, red blood cells, platelets, neutrophils, lymphocytes, monocytes, C-reactive protein (CRP), total protein, albumin, blood urea nitrogen (BUN), and creatinine levels. The inflammatory immune indicators in our study were calculated as follows: CRP-to-albumin ratio (CAR) = CRP/albumin; platelet-to-albumin ratio (PAR) = platelet/albumin; neutrophil-to-albumin ratio (NAR) = neutrophil/albumin; LCR = lymphocyte/CRP; platelet-to-lymphocyte ratio (PLR) = platelet/lymphocyte; neutrophil-to-lymphocyte ratio (NLR) = neutrophil/lymphocyte; systemic-immune-inflammation index (SII) = platelet×neutrophil/lymphocyte; Glasgow prognostic score (GPS), modified GPS (mGPS), and lymphocyte-CRP score (LCS) were determined based on previous reports (11, 12).

### 2.4 Follow-up

After discharge, follow-up was performed by telephone interviews or outpatient visits, which took place 1 month after surgery, then every 3 months for the first 2 years and every 6 months thereafter. OS was defined as the interval from the surgery date to death from any cause or to the last follow-up visit (January 31, 2022).

### 2.5 Statistical analysis

Continuous variables were presented as mean ± SD or median (25th and 75th percentiles) depending on their distribution, categorical variables were presented as numbers and percentages. Student’s t-test, Mann–Whitney U-test and Chi-squared or Fisher’s exact test were used to analyze the differences between groups. The time-dependent receiver operating characteristic (ROC) curve was calculated to compare the predicting ability of systemic inflammation biomarkers at different time points. The optimal cut-off of the inflammation biomarker was determined by the surv_cutpoint function of the R package survminer. Kaplan–Meier method was performed to analyze OS. Several incremental models were established to control for confounding bias, and the Cox proportional hazard regression analyses were used to assess independent risk factors for OS. Restricted cubic spline regression with four knots was used to evaluate the relationship between continuous covariates and OS of patients underwent cardiac surgery in the adjustment models mentioned above. The association of biomarkers and OS in subgroups were analyzed by stratification analysis. Finally, an internal validation was conducted to confirm the effectiveness of the biomarkers by random resampling with a ratio of 70%. A 2-tailed p-value < 0.05 was considered statistically significant. All statistical analyses were performed with SPSS software version 26 (Armonk, NY, USA) and R version 4.1.3 (Vienna, Austria).

## 3 Results

### 3.1 Screening optimal combination of systemic inflammation biomarkers in patients underwent cardiac surgery

According to the inclusion and exclusion criteria, 47 patients were excluded and a total of 820 patients underwent cardiac surgery were enrolled in this study (**Table 1**). The patient group included 542 men and 278 women with a median age of 64 (p25–p75: 57–69) years. Of these patients, 56.7% underwent CABG, 37.7% underwent valve surgery, and 5.6% underwent both CABG and valve surgery.

**Table 1:** Baseline characteristics stratified by LCR.

	Overall (n = 820)	Low LCR (n = 241)	High LCR (n = 579)	p
Age, years	64 (57–69)	65 (59–70)	63 (56–68)	0.002^*^
Sex, male, n (%)	542 (66.1%)	169 (70.1%)	373 (64.4%)	0.116
BMI, kg/m^2^	24.22 (22.34–26.64)	23.88 (21.80–26.66)	24.45 (22.52–26.64)	0.125
Comorbidities, yes, n (%)				
Hypertension	553 (67.4%)	168 (69.7%)	385 (66.5%)	0.371
Diabetes	261 (31.8%)	82 (34.0%)	179 (30.9%)	0.384
Chronic heart failure	63 (7.7%)	23 (9.5%)	40 (6.9%)	0.197
Atrial fibrillation	152 (18.5%)	41 (17.0%)	111 (19.2%)	0.469
Previous myocardial infarction	38 (4.6%)	14 (5.8%)	24 (4.1%)	0.302
Cerebrovascular disease	116 (14.1%)	39 (16.2%)	77 (13.3%)	0.280
Previous PCI	114 (13.9%)	28 (11.6%)	86 (14.9%)	0.223
Smoking, yes, n (%)	284 (34.6%)	88 (36.5%)	196 (33.9%)	0.465
Drinking, yes, n (%)	115 (14.0%)	35 (14.5%)	80 (13.8%)	0.791
LVEF, %	60 (56–64)	60 (53–64)	60 (57–64)	0.002^*^
Surgical Type				0.115
Isolated CABG	465 (56.7%)	150 (62.2%)	315 (54.4%)	
Isolated valve surgery	309 (37.7%)	80 (33.2%)	229 (39.6%)	
CABG + valve surgery	46 (5.6%)	11 (4.6%)	35 (6.0%)	
Operation time, min	213.5 (186.25–245)	213 (191–245)	214 (185–245)	0.620
CPB time, min	81 (56.75–103)	81 (56–103)	81 (57–104)	0.562
Laboratory data				
C-reactive protein, mg/L	3.17 (3.02–4.88)	9.26 (5.65–19.65)	3.02 (3.02–3.23)	<0.001^*^
White blood cells, ×10^9^/L	6.32 (5.16–7.74)	6.77 (5.38–8.41)	6.23 (5.11–7.44)	<0.001^*^
Red blood cells, ×10^12^/L	4.35 ± 0.55	4.15 ± 0.58	4.43 ± 0.52	<0.001^*^
Hemoglobin, g/L	131 (120–143)	123 (112–136.5)	134 (123–144)	<0.001^*^
HCT, %	39.47 ± 4.77	37.69 ± 4.94	40.21 ± 4.50	<0.001^*^
Platelets, ×10^9^/L	203 (162–243)	202 (159–255.5)	203 (163–240)	0.332
Neutrophil percentage, %	62.11 ± 9.85	67.91 ± 9.41	59.69 ± 8.98	<0.001^*^
Lymphocytes, ×10^9^/L	1.69 (1.35–2.11)	1.39 (1.06–1.80)	1.82 (1.44–2.21)	<0.001^*^
Monocytes, ×10^9^/L	0.43 (0.33–0.55)	0.46 (0.36–0.63)	0.41 (0.33–0.51)	<0.001^*^
Total protein, g/L	68.8 (65.0–72.0)	68.8 (64.1–72.0)	68.8 (65.0–72.2)	0.504
Albumin, g/L	41 (39–44)	40 (37–43)	42 (40–44)	<0.001^*^
BUN, μmol/L	5.90 (4.81–7.30)	5.93 (4.75–7.40)	5.90 (4.89–7.20)	0.646
Creatinine, μmol/L	75.5 (63.0–89.0)	77.0 (63.6–91.8)	74.6 (63.0–88.2)	0.079

LCR, lymphocyte-to-C-reactive protein ratio; BMI, body mass index; PCI, percutaneous coronary intervention; LVEF, left ventricular ejection; CABG, coronary artery bypass grafting; CPB, cardiopulmonary bypass; HCT, hematocrit; BUN, blood urea nitrogen.

*p < 0.05.

**Figure 1:**
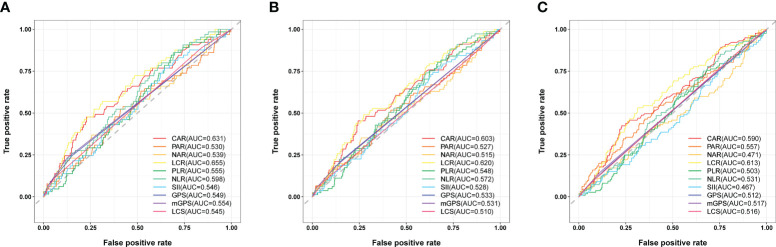
Comparison the effectiveness of systemic inflammation biomarkers in predicting the prognosis after cardiac surgery. **(A)** ROC curve analysis for survival prediction at 1-year. **(B)** ROC curve analysis for survival prediction at 2-year. **(C)** ROC curve analysis for survival prediction at 3-year.

Time-dependent ROC curve at different time points was conducted to compare the effectiveness of the ten systemic inflammation biomarkers in predicting the OS of patients underwent cardiac surgery (**Figure 1**). The area under the ROC of LCR was 0.655, 0.620 and 0.613 at 1-, 2- and 3-year, respectively. The effectiveness of LCR was also compared with CRP alone, as well as with different populations of white blood cells (**Supplementary Figure 1**). Among these systemic inflammation biomarkers, LCS (0.619 [0.494, 0.743]) had the highest C-index for OS after cardiac surgery, followed by LCR (0.611 [0.548, 0.674]) (**Table 2**). Based on these findings, it appears that LCR may serve as a favorable indicator for predicting the prognosis of cardiac surgery. The optimal cut-off of LCR was 0.35, determined by the standardized log-rank statistic based on the survival status (**Supplementary Figure 2**). Thus, 241 and 579 patients were identified as low and high LCR respectively. The two groups were comparable in terms of sex, body mass index (BMI), comorbidities and lifestyle. Although the age and left ventricular ejection (LVEF) of the two groups were quite close, patients with low LCR were elder (65 [59–70] vs 63 [56–68] years, p = 0.002), and had lower LVEF (60 [53–64] vs 60 [57–64] %, p=0.002). Besides, patients with low LCR had higher white blood cells (p<0.001), neutrophil percentage (p<0.001), monocytes (p<0.001) and lower red blood cells (p<0.001), hemoglobin (p<0.001), hematocrit (HCT, p<0.001), albumin (p<0.001).

**Table 2:** The C-index of systemic inflammation biomarkers for OS in patients underwent cardiac surgery.

	C-index	95% CI
CAR	0.555	0.497, 0.614
PAR	0.538	0.476, 0.601
NAR	0.499	0.438, 0.560
LCR	0.611	0.548, 0.674
PLR	0.486	0.426, 0.545
NLR	0.509	0.455, 0.564
SII	0.461	0.401, 0.521
GPS	0.570	0.439, 0.701
mGPS	0.574	0.435, 0.713
LCS	0.619	0.494, 0.743

CAR, CRP-to-albumin ratio; PAR, platelet-to-albumin ratio; NAR, neutrophil-to-albumin ratio; LCR, lymphocyte-to-CRP ratio; PLR, platelet-to-lymphocyte ratio; NLR, neutrophil-to-lymphocyte ratio; SII, systemic-immune-inflammation index; GPS, Glasgow prognostic score; mGPS, modified Glasgow prognostic score; LCS, lymphocyte-CRP score.

### 3.2 LCR effectively predicted the clinical outcomes after cardiac surgery

**Table 3** presented the short-term outcomes of patients underwent cardiac surgery, with 367 (44.8%) experiencing postoperative complications. Low LCR was associated with a higher incidence of total complications (50.6% vs 42.3%, p = 0.029) and in-hospital mortality (9.1% vs 3.3%, p<0.001), with corresponding increases in hospitalization expenses (135145 [116721–161630] vs 128244 [111187–152505] Yuan, p = 0.009).

**Table 3:** Details for short-term outcomes.

	Overall (n = 820)	Low LCR (n = 241)	High LCR (n = 579)	p
Total Complications	367 (44.8%)	122 (50.6%)	245 (42.3%)	0.029*
Pneumonia	31 (3.8%)	11 (4.6%)	20 (3.5%)	0.448
Delirium	26 (3.2%)	8 (3.3%)	18 (3.1%)	0.875
Poor wound healing (no debridement)	20 (2.4%)	7 (2.9%)	13 (2.2%)	0.577
Pleural effusion	120 (14.6%)	40 (16.6%)	80 (13.8%)	0.305
Poor wound healing need debridement	21 (2.6%)	6 (2.5%)	15 (2.6%)	0.933
Reoperation	8 (1.0%)	2 (0.8%)	6 (1.0%)	1.000
Stroke	4 (0.5%)	1 (0.4%)	3 (0.5%)	1.000
Low cardiac output syndrome	29 (3.5%)	10 (4.1%)	19 (3.3%)	0.540
Respiratory failure	59 (7.2%)	13 (5.4%)	46 (7.9%)	0.198
MODS	8 (1.0%)	2 (0.8%)	6 (1.0%)	1.000
In-hospital mortality	41 (5.0%)	22 (9.1%)	19 (3.3%)	<0.001*
Postoperative hospital stay, day	10 (8–13)	10 (9–14)	10 (8–13)	0.272
Prolonged intensive care stay(>5d)	122 (14.9%)	41 (17.0%)	81 (14.0%)	0.268
Indwelling drainage tube time, day	3 (3–4)	3 (3–4)	3 (3–4)	0.331
Hospitalization expenses, Yuan	130229 (112779–155771)	135145 (116721–161630)	128244 (111187–152505)	0.009*
30 days readmission	48 (5.9%)	16 (6.6%)	32 (5.5%)	0.537

LCR, lymphocyte-to-C-reactive protein ratio; MODS, multiple organ dysfunction syndrome.

*p < 0.05.

**Figure 2:**
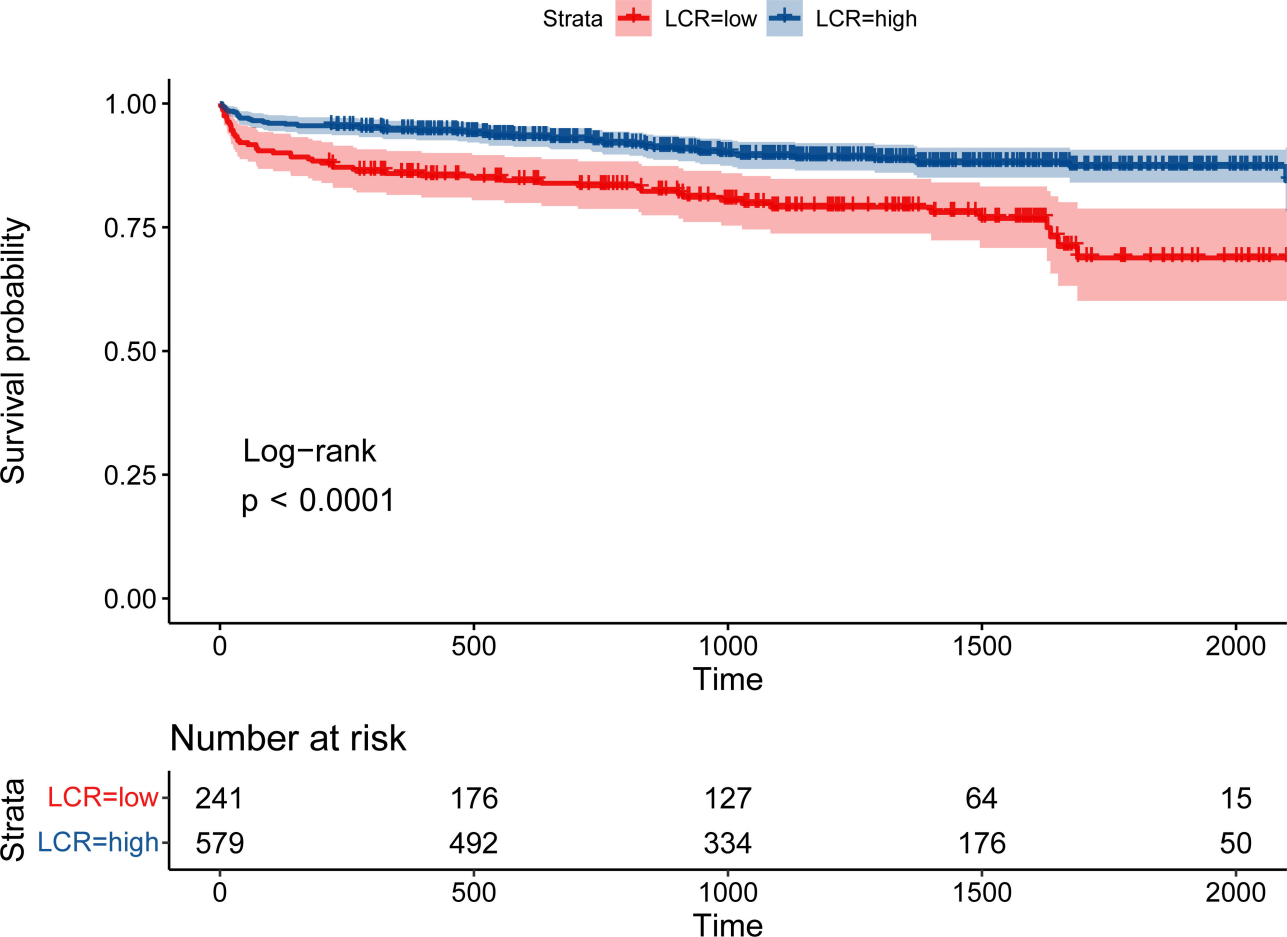
Kaplan–Meier curve stratified by LCR in patients underwent cardiac surgery.

**Figure 3:**
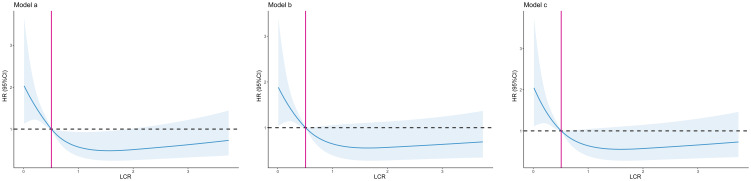
The association between LCR and all-cause mortality after cardiac surgery. Model a: No adjusted. Model b: Adjusted for age, sex, BMI, surgical type. Model c: Adjusted for age, sex, BMI, surgical type, hypertension, diabetes, chronic heart failure, cerebrovascular disease, operation time.

During the follow-up period (median 3.36 [2.18–4.51] years), 113 (13.8%) patients died. Patients with low LCR had a significantly higher mortality rate than those with high LCR (21.6% vs 10.5%, log-rank p <0.001, **Figure 2**). Restricted cubic spline regression revealed a significant nonlinearity between LCR and the OS of patients underwent cardiac surgery (**Figure 3**). Patients with lower LCR may experience a worse prognosis, and this trend persisted across all three models. Low LCR was defined as an independent risk factor for poor OS through multivariate Cox regression (**Table 4**). When LCR was treated as a continuous variable, the decrease based on standard deviation may not reflect the increased risk of mortality (p = 0.556). When LCR was divided into a categorical variable, low LCR demonstrated a close relationship with a poor prognosis in model c (HR = 0.468, 95% CI = 0.319–0.687, p<0.001). When LCR was divided into quartiles, the risk of poor prognosis gradually decreased compared to the lowest quintile Q1 (<0.29) and the HRs for OS in model c were 0.597 (95% CI =0.369–0.967), 0.507 (95% CI = 0.300–0.857) and 0.389 (95% CI = 0.220–0.686), respectively. When patients were stratified by age, sex, BMI, comorbidities, drinking status, smoking status, LVEF and surgical type, stratification analysis showed that the relationship between low LCR and poor OS remained stable in most of the subgroups (**Supplementary Figure 3**). None of the covariates had an interaction with low LCR (all p for interaction >0.050).

**Table 4:** Association between LCR and overall survival of patients underwent cardiac surgery.

LCR	Model a	p value	Model b	p value	Model c	p value
Continuous (per SD)	0.879 (0.681–1.136)	0.325	0.915 (0.737–1.137)	0.423	0.931 (0.735–1.180)	0.556
Cutoff value		<0.001*		<0.001*		<0.001*
C1 (≤0.35)	Ref		Ref		Ref	
C2 (>0.35)	0.442 (0.305–0.640)		0.486 (0.333–0.708)		0.468 (0.319–0.687)	
Quartiles						
Q1 (<0.29)	Ref		Ref		Ref	
Q2 (0.29–0.51)	0.630 (0.395–1.005)	0.052	0.639 (0.398–1.025)	0.063	0.597 (0.369–0.967)	0.036*
Q3 (0.51–0.71)	0.464 (0.277–0.775)	0.003*	0.510 (0.302–0.859)	0.011*	0.507 (0.300–0.857)	0.011*
Q4 (≥0.71)	0.368 (0.212–0.638)	<0.001*	0.425 (0.243–0.741)	0.003*	0.389 (0.220–0.686)	0.001*
p for trend		0.001*		0.008*		0.004*

Model a: No adjusted.

Model b: Adjusted for age, sex, BMI, surgical type.

Model c: Adjusted for age, sex, BMI, surgical type, hypertension, diabetes, chronic heart failure, cerebrovascular disease, operation time.

^*^p < 0.05.

### 3.3 Randomized internal validation for the prognostic value of LCR

Random resampling with a ratio of 70% was performed to simulate outsource validation cohort and 574 patients were enrolled (**Supplementary Table 1**). Consistent with previous findings, Kaplan–Meier curve showed that LCR can effectively stratify the prognosis of patients underwent cardiac surgery in validation cohort (**Supplementary Figure 4**). Additionally, restricted cubic spline regression demonstrated a reverse L-shaped relationship between LCR and HRs of OS across all three models (**Supplementary Figure 5**). **Supplementary Table 2** presented the prognostic value of LCR when analyzed as a continuous variable, categorical variable or quartiles. Patients with low LCR have poorer OS than those with high LCR in model c (HR = 0.445, 95% CI = 0.279–0.709, p<0.001).

## 4 Discussion

In this study, we conducted a comprehensive comparative analysis to investigate the prognostic value of multiple systemic inflammation biomarkers in patients underwent cardiac surgery. Time-dependent ROC curve at different time points and C-index were conducted to compare the effectiveness of these ten systemic inflammation biomarkers in predicting the OS of patients underwent cardiac surgery. The effectiveness of LCR was also compared with CRP alone, as well as with different populations of white blood cells. To our knowledge, this is the first study to identify LCR as a promising systemic inflammation biomarker for predicting prognosis after cardiac surgery. Patients with low LCR had a higher risk of postoperative complications and incurred higher hospitalization expenses. Moreover, LCR was deemed as an independent risk factor of OS after cardiac surgery, which has been further confirmed in the randomized internal validation.

Patients underwent cardiac surgery are likely to be in a state of preoperative chronic inflammation triggered by cardiac disease itself. Atherosclerosis is an inflammatory disease, with chronic inflammation of the arterial wall causing slow formation of lesions and progressive narrowing of arterial lumen (7, 8). Degenerative valve disease involves inflammatory factors that affect the interaction between valvular endothelial cells, valvular interstitial cells and extracellular matrix, leading to destruction of normal structure-function correlations (9). Valve ageing and disease brings abnormal blood flow patterns, which damages the endothelium and produces chronic inflammation. Laura et al. reported that patients with aortic stenosis have reduced levels of complement C4, verifying that a pro-inflammatory state exists in calcific aortic valve disease (13). In addition, traditional risk factors of CVD, such as hypertension, diabetes and hyperlipidemia, trigger vascular inflammatory response and lead to plaque disruption and the occurrence of major adverse cardiac and cerebrovascular event (MACCE) (14–16). Elevated proinflammatory cytokine levels have been detected in early deterioration of cardiac function, which is more sensitive than neurohormonal biomarkers (17). Increasing circulating inflammatory cytokines, such as tumor necrosis factor (TNF)-α, interleukin (IL)-1 and IL-6, has been observed in patients with heart failure, which may predict clinical outcomes, but the application of these cytokines is limited by the low circulating volumes and expensive assays (18–21). All this evidence points to a strong relationship between CVD and inflammation.

Due to the chronic inflammation present in patients underwent cardiac surgery, inflammation-induced catabolic stress exacerbates the patients’ depletion and worsens their nutritional status, ultimately leading to poor clinical outcomes. Therefore, there is a pressing need for simple and readily available markers to predict the clinical outcomes of patients underwent cardiac surgery. Some single inflammatory factors appear to be available to predict clinical outcomes (22, 23), which is consistent with our study. However, the predictive value of individual inflammatory factors, such as CRP and white blood cells, was mostly confined to 1-year OS, which declined rapidly over time. Moreover, the predictive value of a single inflammatory factor is not as effective as a combination of multiple factors (24–26). Serum systemic inflammation biomarkers are derived from a simple calculation of the existing preoperative examination and offer the advantage of convenience and low cost. Among the 10 biomarkers analyzed in this study, LCR was the most effective tool for the prediction of clinical outcomes after cardiac surgery.

LCR is derived from the ratio of lymphocyte count to CRP. Lymphocytes play a key role in the regulation of the immune response, including both innate and adaptive immunity (27). The lymphocyte count reflects the immune-nutrition status of the body. Since activated effector lymphocytes primarily rely on glycolysis for their high energy requirements, they are particularly sensitive to poor nutritional status (28, 29). The number and function of lymphocytes are altered in malnutrition. Lymphopenia demonstrates low immune function and increases susceptibility to infection, while infections worsen nutritional status by increasing the demand for nutrients and decreasing appetite. Malnutrition further increase susceptibility to infection, creating a vicious cycle and resulting in adverse effects on the clinical outcomes of patients. CRP is widely used to determine the presence of acute inflammation in clinical practice (30). Muscle and visceral protein breakdown increase and they are used to provide energy during the acute phase, thought to exacerbate malnutrition (31). Studied have reported that CRP is associated with the prognosis of CVD, such as atherosclerotic disease and aortic valve disease, suggesting an active role in the pathophysiology of CVD (32–34). Meanwhile, CRP activates the classical complement pathway and mediates recognition and phagocytosis (35). Therefore, LCR, which combines CRP with lymphocyte count, comprehensively reflects the immunology, nutrition and inflammatory state of the host.

Current researches in LCR primarily focus on solid tumor and sarcoma (4–6), COVID-19 (36) and hemiarthroplasty (37). Only a few studies have reported its predictive value in CVD, such as STEMI (38) or patients underwent heart transplantation (39). Our study is the first to report the prognostic value of LCR in predicting the clinical outcomes after cardiac surgery, suggesting the potential benefit of anti-inflammatory intervention in improving the prognosis of cardiac surgery. A meta-analysis demonstrated that the impact of prophylactic steroids on clinical outcomes after cardiac surgery is still controversial (40). Statins and other lipid-lowering agents have anti-inflammatory effects, and current evidence has provided enough facts for their beneficial effects after cardiac surgery (41, 42). Novel anti-inflammatory medications in CVD also shows the potential benefit for cardiovascular risk reduction (43).

This study has several limitations. As a retrospective, single-institutional study, no external data was used to validate the prediction model. So we require further validation of multicenter, multiethnic studies in the future. However, our study has the strength of having complete biomarker data since every patient has undergone preoperative blood tests. Besides, all systemic inflammation markers were detected only once before surgery. We will focus on the impact of trajectory changes of these biomarkers in our further study.

## 5 Conclusions

The present study has confirmed that LCR holds considerable potential as a representative systemic inflammation biomarker for prognosticating outcomes after cardiac surgery. Patients with low LCR had a higher risk of postoperative complications and worse overall survival. Detecting low LCR levels could prove beneficial for surgeons in identifying patients at higher risk and tailoring perioperative management strategies accordingly.

## Data availability statement

The raw data supporting the conclusions of this article will be made available by the authors, without undue reservation.

## Ethics statement

The studies involving humans were approved by the Ethics Committee of Shanghai Tenth People’s Hospital. The studies were conducted in accordance with the local legislation and institutional requirements. The participants provided their written informed consent to participate in this study.

## Author contributions

ZL, WZ and ZS designed the study. PZ, GZ and YZ collected the data. ZL and WZ did the analysis and interpretation of data. ZS wrote and revised the article. All authors contributed to the article and approved the submitted version.
