# Supplementary material for: Comprehensive comparative analysis of the prognostic impact of systemic inflammation biomarkers for patients underwent cardiac surgery
Source: Front Immunol. 2023 Aug 14;14:1190380. doi: 10.3389/fimmu.2023.1190380 (PMC10461628; doi:10.3389/fimmu.2023.1190380)
Supplement: Supplementary file 1 [file DataSheet_1.docx]

Supplementary Material

**Supplementary Table 1**

Baseline characteristics at validation cohort

|  | Validation cohort  (n = 574) |
| --- | --- |
| Age, years | 64 (57–69) |
| Sex, male, n (%) | 385 (67.1%) |
| BMI, kg/m^2^ | 24.35 (22.38–26.61) |
| Comorbidities, yes, n (%) |  |
| Hypertension | 387 (67.4%) |
| Diabetes | 176 (30.7%) |
| Chronic heart failure | 45 (7.8%) |
| Atrial fibrillation | 102 (17.8%) |
| Previous myocardial infarction | 27 (4.7%) |
| Cerebrovascular disease | 76 (13.2%) |
| Previous PCI | 80 (13.9%) |
| Smoking, yes, n (%) | 199 (34.7%) |
| Drinking, yes, n (%) | 78 (13.6%) |
| LVEF, % | 60 (55–64) |
| Surgical Type |  |
| Isolated CABG | 319 (55.6%) |
| Isolated valve surgery | 218 (38.0%) |
| CABG + valve surgery | 37 (6.4%) |
| Operation time, min | 214 (189–246) |
| CPB time, min | 82 (60–104) |
| Laboratory data |  |
| C-reactive protein, mg/L | 3.17 (3.02–5.03) |
| White blood cells, ×10^9^/L | 6.34 (5.14–7.79) |
| Red blood cells, ×10^12^/L | 4.35 ± 0.54 |
| Hemoglobin, g/L | 131 (119–143) |
| HCT, % | 39.47 ± 4.67 |
| Platelets, ×10^9^/L | 203 (161–244) |
| Neutrophil percentage, % | 62.32 ± 9.90 |
| Lymphocytes, ×10^9^/L | 1.68 (1.35–2.11) |
| Monocytes, ×10^9^/L | 0.43 (0.34–0.55) |
| Total protein, g/L | 68.8 (65.0–72.0) |
| Albumin, g/L | 41 (39–44) |
| BUN, μmol/L | 5.86 (4.80–7.30) |
| Creatinine, μmol/L | 75.1 (62.5–89.0) |

LCR, lymphocyte-to-C-reactive protein ratio; BMI, body mass index; PCI, percutaneous coronary intervention; LVEF, left ventricular ejection; CABG, coronary artery bypass grafting; CPB, cardiopulmonary bypass; HCT, hematocrit; BUN, blood urea nitrogen.

**Supplementary Table 2**

Association between LCR and overall survival of patients underwent cardiac surgery at validation cohort

| LCR | Model a | p value | Model b | p value | Model c | p value |
| --- | --- | --- | --- | --- | --- | --- |
| Continuous (per SD) | 0.960 (0.752–1.224) | 0.741 | 0.981 (0.820–1.175) | 0.838 | 1.006 (0.826–1.224) | 0.955 |
| Cutoff value |  | <0.001* |  | 0.001* |  | 0.001* |
| C1 (≤0.35) | Ref |  | Ref |  | Ref |  |
| C2 (>0.35) | 0.415 (0.266–0.647) |  | 0.470 (0.298–0.740) |  | 0.445 (0.279–0.709) |  |
| Quartiles |  |  |  |  |  |  |
| Q1 (<0.29) | Ref |  | Ref |  | Ref |  |
| Q2 (0.29–0.51) | 0.446 (0.245–0.811) | 0.008* | 0.424 (0.231–0.777) | 0.006* | 0.394 (0.212–0.732) | 0.003* |
| Q3 (0.51–0.71) | 0.449 (0.247–0.816) | 0.009* | 0.504 (0.273–0.928) | 0.028* | 0.490 (0.263–0.911) | 0.024* |
| Q4 (≥0.71) | 0.379 (0.200–0.721) | 0.003* | 0.462 (0.240–0.886) | 0.020* | 0.428 (0.219–0.836) | 0.013* |
| p for trend |  | 0.003* |  | 0.011* |  | 0.006* |

Model a: No adjusted

Model b: Adjusted for age, sex, BMI, surgical type

Model c: Adjusted for age, sex, BMI, surgical type, hypertension, diabetes, chronic heart failure, cerebrovascular disease, operation time

* p<0.05

**Supplementary Figure 1**

Comparison the effectiveness of LCR, CRP and different populations of white blood cells (WBC) in predicting the prognosis after cardiac surgery. (A) ROC curve analysis for survival prediction at 1-year. (B) ROC curve analysis for survival prediction at 2-year. (C) ROC curve analysis for survival prediction at 3-year.


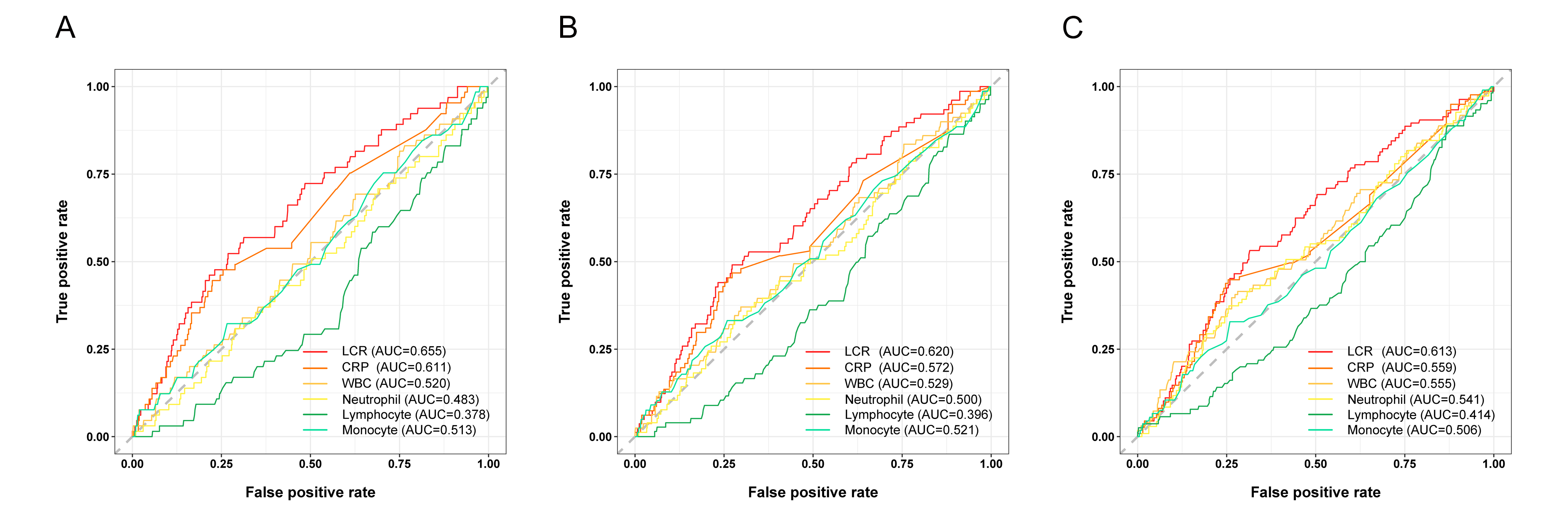


**Supplementary Figure 2**

The optimal cut-off of LCR determined by “surv_cutpoint” function


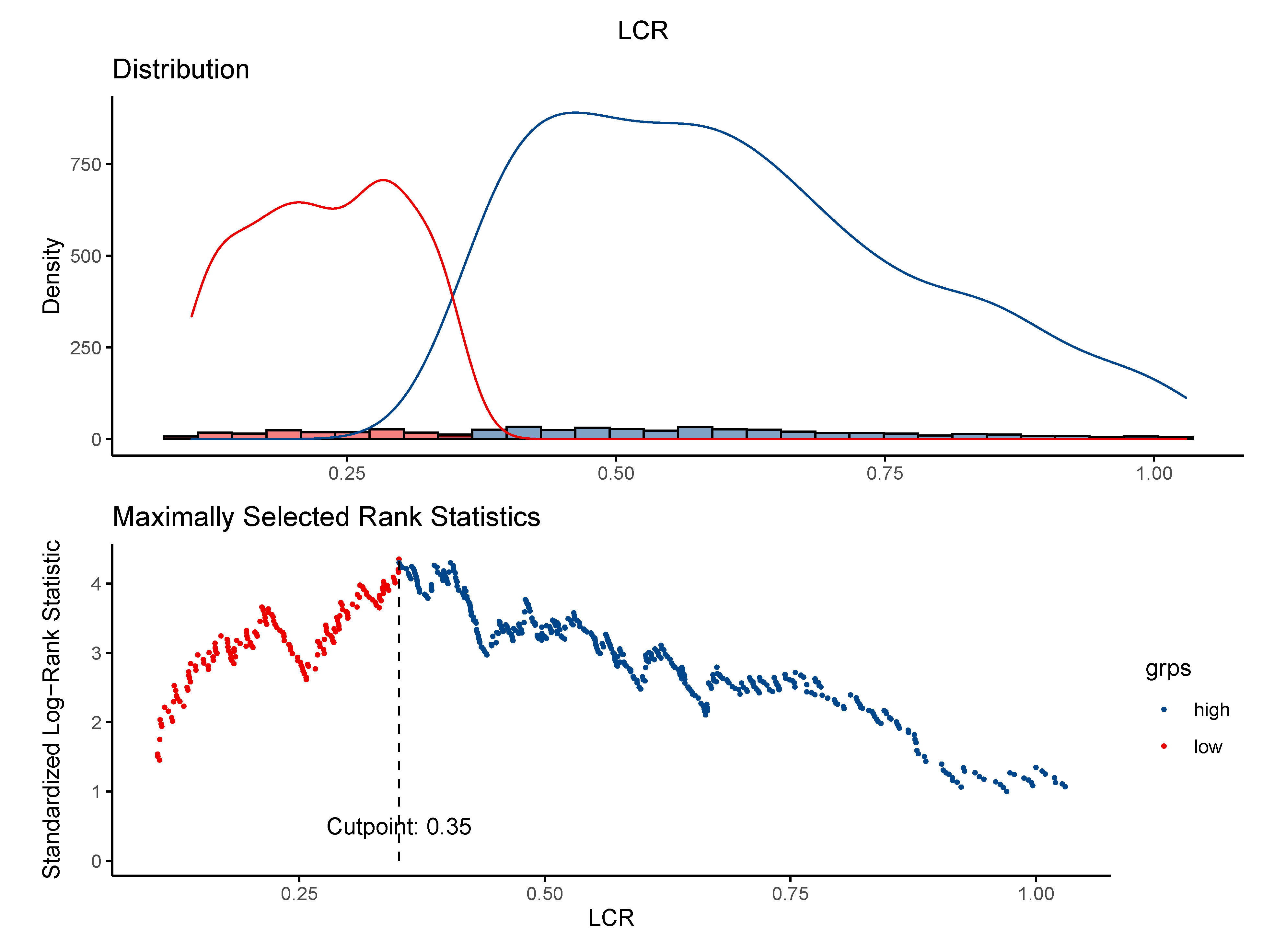


**Supplementary Figure 3**

The association between LCR and hazard ratios of overall survival in various subgroups

**
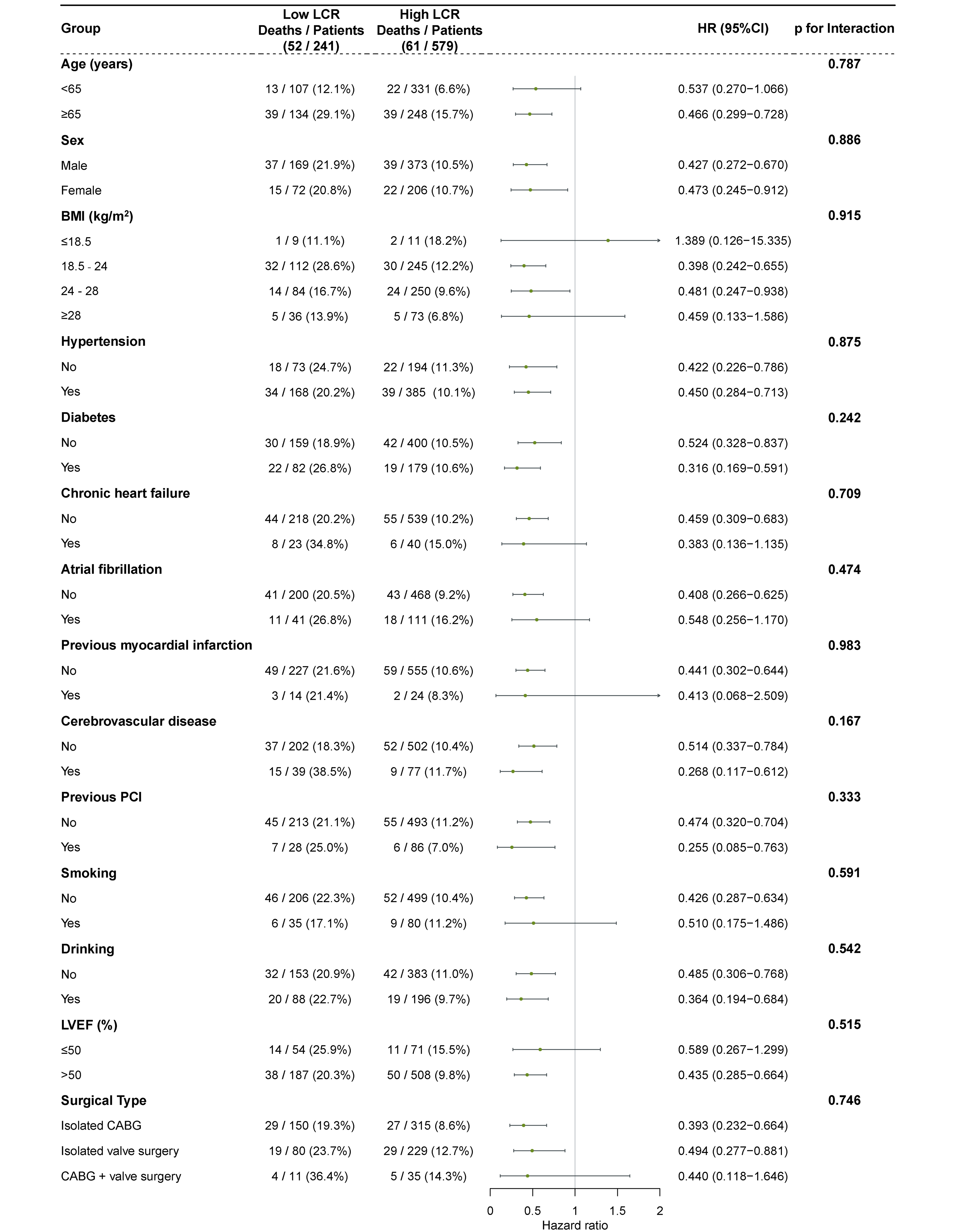
**

**Supplementary Figure 4**

Kaplan–Meier curve stratified by LCR after cardiac surgery in validation cohort

**
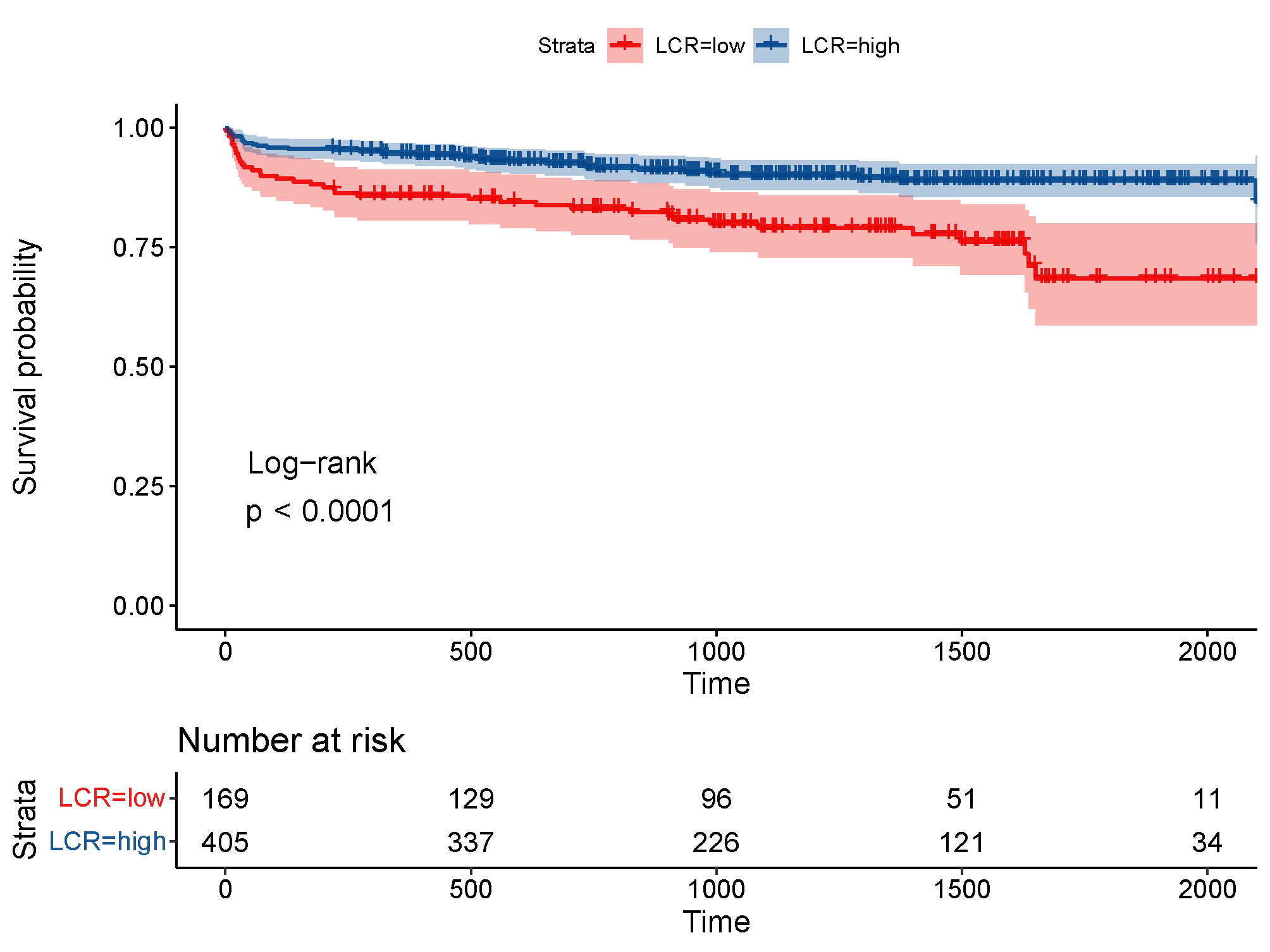
**

**Supplementary Figure 5**

The association between LCR and all-cause mortality after cardiac surgery in validation cohort. Model **a** No adjusted. Model **b** Adjusted for age, sex, BMI, surgical type. Model **c** Adjusted for age, sex, BMI, surgical type, hypertension, diabetes, chronic heart failure, cerebrovascular disease, operation time

**
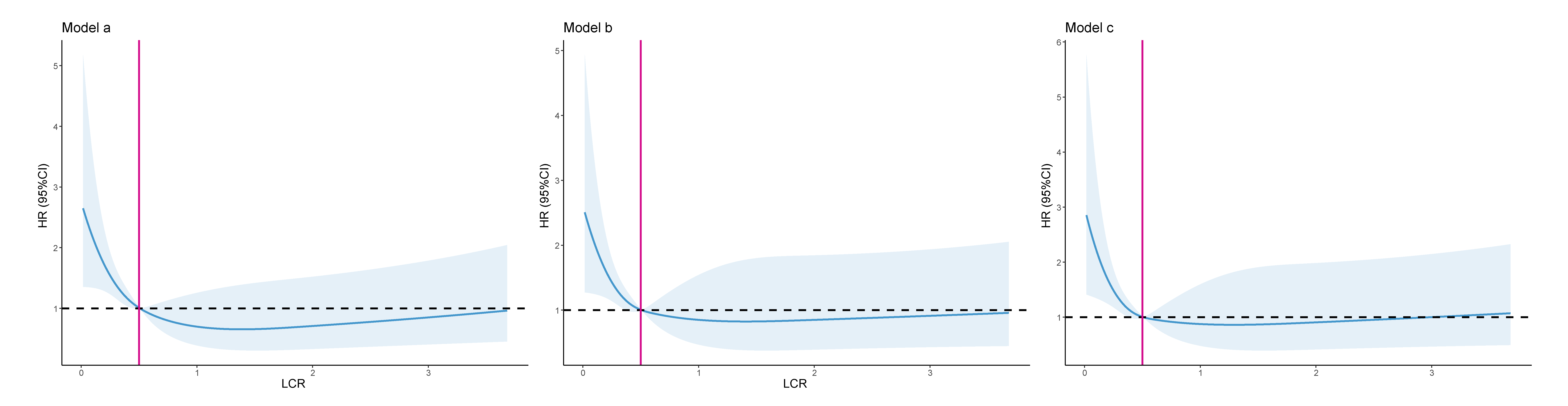
**
